# Simultaneous Lymphoepithelioma-Like and Plasmacytoid Subtypes of Urothelial Carcinoma, Along With Prostatic Adenocarcinoma With Clinical Follow-Up

**DOI:** 10.1155/crip/9068792

**Published:** 2025-08-21

**Authors:** Siddharth Venkatesh, John S. Costanza, Bettye Cox, Chris Finch, Ya Xu

**Affiliations:** ^1^Baylor College of Medicine, Houston, Texas, USA; ^2^Department of Pathology & Immunology, Baylor College of Medicine, Houston, Texas, USA; ^3^Department of Pathology & Laboratory Medicine, Ben Taub Hospital, Harris Health System, Houston, Texas, USA; ^4^Department of Surgery, Pathology Service, HCA Houston Healthcare Clear Lake, Webster, Texas, USA

**Keywords:** lymphoepithelioma-like urothelial carcinoma (LELUC), plasmacytoid urothelial carcinoma (PUC), prostatic adenocarcinoma, urinary bladder

## Abstract

Lymphoepithelioma-like urothelial carcinoma (LELUC) and plasmacytoid urothelial carcinoma (PUC) are rare subtypes. We report a case of simultaneous urothelial carcinoma composed of LELUC and PUC subtypes, along with prostatic adenocarcinoma, with successful clinical management by immunotherapy. The patient, a 54-year-old man with a 40 pack-year smoking history, presented with gross hematuria and dysuria. Imaging revealed focal bladder wall thickening. The patient underwent transurethral resection of bladder tumor (TURBT), followed by cystoprostatectomy. The TURBT revealed LELUC, with muscle invasion. The subsequent cystoprostatectomy specimen displayed a 6.0 cm ulcerative mass, which had focal penetration through the urinary bladder wall. Microscopically, the tumor consisted of sheets of enlarged and pleomorphic tumor cells, mixed with a lymphoplasmacytic infiltrate. Focal plasmacytoid and occasional signet ring cell-like morphologies were observed. Rare tumor cells showed positivity for GATA-3 and p63 immunostains, while the plasmacytoid tumor cells exhibited loss of E-cadherin expression. Additionally, adenocarcinoma of the prostate was present, with a Gleason score of 3 + 3, involving 2% of the prostate tissue. The diagnoses of LELUC, comprising 95% of the tumor, PUC, comprising 5%, and prostatic adenocarcinoma were made. Molecular studies revealed a high tumor mutational burden, and the tumor exhibited PD-L1 expression. The patient received adjuvant immunotherapy with Pembrolizumab and showed no evidence of disease for 3 years up to the time of this report. Morphologic recognition of the various subtypes of urothelial carcinoma, supported by immunohistochemistry, is essential for the proper clinical management of patients. A search of the literature on PubMed revealed no similar cases.

## 1. Introduction

Urinary bladder cancers are composed of approximately 75% pure urothelial carcinoma (UC) and 25% histologic variants [1]. All histological subtypes of invasive UC are considered high-grade tumors and have significant diagnostic, prognostic, and therapeutic implications. Some histologic variants of UC can be found admixed with conventional and other subtypes. Accurate diagnosis facilitates risk stratification, informs prognosis, and guides treatment decisions, particularly since treatments for variant histologic subtypes may differ from those for pure UC [1, 2]. Here, we present an exceptionally rare case involving a combination of two uncommon variants: lymphoepithelioma-like urothelial carcinoma (LELUC) and plasmacytoid urothelial carcinoma (PUC), alongside prostate adenocarcinoma. Additionally, we will discuss the latest data on the diagnosis and differential diagnosis of UC subtypes.

## 2. Case Presentation

### 2.1. Clinical Presentation

A 54-year-old man with a 40 pack-year smoking history presented to the emergency department with symptoms of urinary retention, gross hematuria, and dysuria. Computerized tomography (CT) imaging revealed focal thickening of the bladder wall at the right posterolateral wall. Cystoscopy identified a large bladder tumor on the right lateral wall, encompassing the entire trigone. The patient underwent transurethral resection of bladder tumor (TURBT) and subsequent radical cystoprostatectomy.

### 2.2. Histopathologic Findings

**Figure 1:**
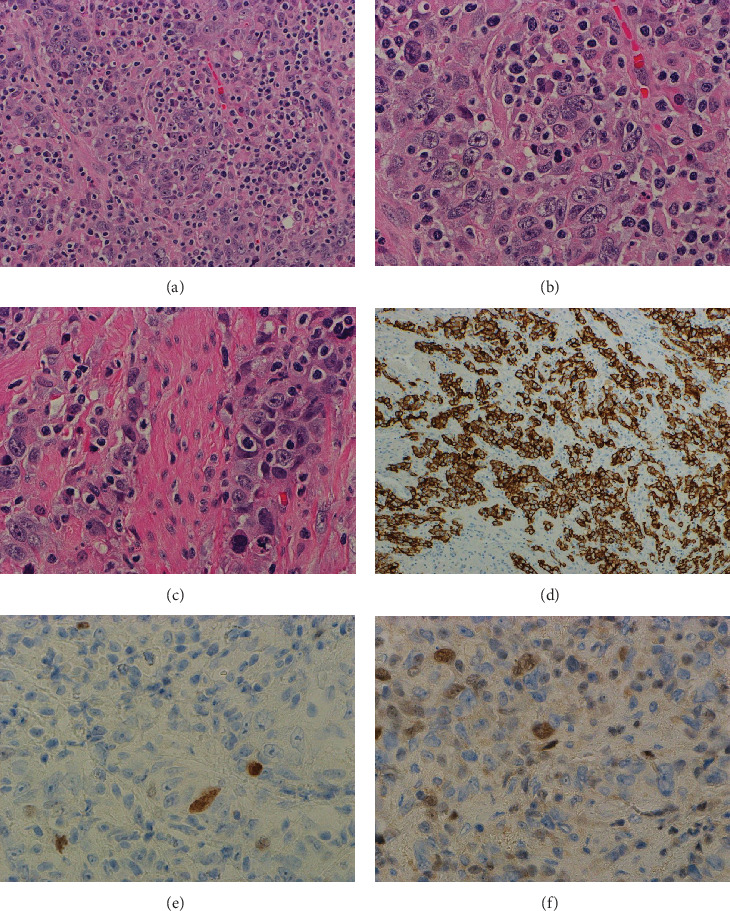
Morphological features of lymphoepithelioma-like urothelial carcinoma (LELUC) component in cystoprostatectomy specimen. There are sheets of undifferentiated and pleomorphic tumor cells in the background of lymphoplasmacytic infiltrate ((a) 200X; (b) 400X). The tumor invades into muscularis propria ((c) 400X). The tumor cells are positive for Pan-CK ((d) 100X) with rare tumor cells positive for p63 ((e) 400X) and GATA3 ((f) 400X).

Histological examination of the TURBT revealed sheets of undifferentiated cells with a background of small lymphocytes, resembling the lymphoepithelioma-like subtype. The Epstein–Barr virus (EBV) in situ hybridization was performed, yielding negative results. The specimen of cystoprostatectomy revealed a 6.0-cm tan-red ulcerative mass grossly identified in the urinary bladder, with focal abutment against the perivesical adipose tissue of the dome. Microscopically, a high-grade UC was found to extensively involve the urothelial mucosa, lamina propria, and muscularis propria. The tumor was arranged in sheets and comprised undifferentiated cells with large pleomorphic nuclei, prominent nucleoli, indistinct cytoplasmic borders, and a syncytial appearance (Figures 1a, 1b, and 1c). Mitotic figures were easily identified. The background exhibited a prominent lymphocytic infiltrate, with the presence of plasma cells, neutrophils, and eosinophils. Lymphovascular invasion and rare perineural invasion were also identified.

**Figure 2:**
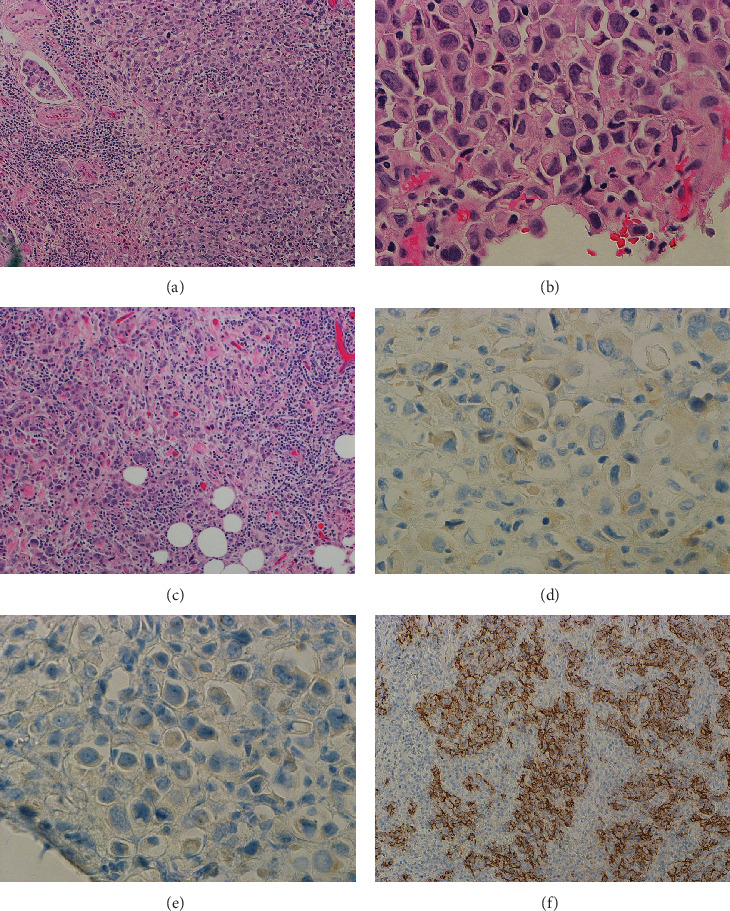
Morphological features of plasmacytoid urothelial carcinoma (PUC) component compared to LELUC in cystoprostatectomy specimen. An area of plasmacytoid tumor cells in sheets and lymphovascular invasion is present ((a) 100X). The tumor cells have eccentric nuclei with plasmacytoid and signet ring cell-like appearances ((b) 400X). The tumor extends into perivesical soft tissue ((c) 100X). The tumor is negative for CD138 ((d) 400X) and displays loss of E-Cadherin expression ((e) 400X). LELUC has E-Cadherin membranous expression ((f) 100X).

A small component of plasmacytoid carcinoma was observed arranged in sheets within the mucosa, penetrating through the muscularis propria, and focally present in the perivesical soft tissue of the dome and ureteral mucosa (Figures 2a, 2b, and 2c). Some clusters of tumor cells were surrounded by retraction spaces. These tumor cells exhibited eccentric nuclei and glassy eosinophilic cytoplasm, with some displaying a signet ring-like appearance. The identification of the plasmacytoid carcinoma component invading the perivesical soft tissue on microscopic examination resulted in a pathologic stage of pT3a.

The prostate showed adenocarcinoma (Gleason score 3 + 3), involving 2% of prostate tissue, with the greatest dimension of the dominant nodule being 12 mm (organ-confined, pT2). All margins are negative in the radical cystoprostatectomy specimen. Abdominal and pelvic lymph node dissection showed that all 52 lymph nodes are negative for metastatic carcinoma.

### 2.3. Immunohistochemistry (IHC)

IHC was carried out using vendor-supplied, prediluted primary antibodies. Antigen retrieval was performed using a standardized protocol on the BenchMark Ultra automated IHC platform. Specifically, Cell Conditioning Solution 1 (CC1), a Tris-based buffer preformulated by Ventana, served as the retrieval reagent. This solution was applied at elevated temperature (approximately 100°C) within the automated stainer to facilitate effective epitope unmasking prior to antibody incubation. The antibodies utilized in this study are presented in Table 1.

IHC revealed positivity for pan-cytokeratin (PanCK) in the tumor, with rare tumor cells showing positivity for GATA3 and p63. The predominant tumor component (95%) was identified as LELUC (Figures 1d, 1e, and 1f).

The small plasmacytoid tumor component described above showed loss of E-cadherin expression and was negative for CD138 by IHC (Figure 2d,e). The predominant tumor component, LELUC, exhibited membranous expression of E-cadherin (Figure 2f). These findings support the diagnosis of the PUC component, an aggressive subtype of UC, comprising approximately 5% of the tumor.

### 2.4. Molecular Study

The tissue from TURBT was sent for molecular studies by next-generation sequencing (NGS). Comprehensive molecular profiling was performed as an external reference test, with analysis and interpretation completed by TEMPUS. According to the report, the assay utilized was the Tempus xT panel (Version 4), a tailored genomic testing platform encompassing 648 cancer-related genes. This assay identifies single nucleotide alterations, small insertions and deletions, copy number changes, and structural rearrangements through hybrid capture-based NGS, employing proprietary probes developed by Integrated DNA Technologies (IDT). A summary of the key genetic alterations identified in this case is provided in Table 2.

**Figure 3:**
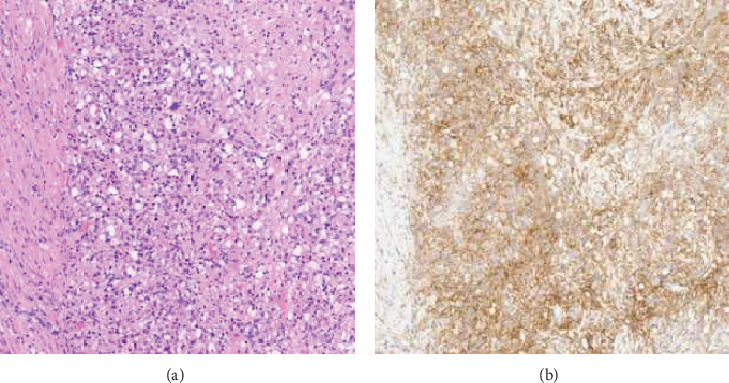
Positive PD-L1 expression in the urothelial carcinoma on the TURBT specimen. (a) There are sheets of tumor cells on H&E stain. (b) Immunohistochemistry demonstrates positive PD-L1 expression, with a tumor proportion score (TPS) of 80% and a combined positive score (CPS) of 90%.

Importantly, NGS revealed a high tumor mutational burden (TMB) of 18.9 mutations per megabase, placing it in the 95th percentile. Additionally, IHC demonstrated positive PD-L1 expression, with a tumor proportion score (TPS) of 80% and a combined positive score (CPS) of 90%, using the DAKO PD-L1 22C3 clone (Figure 3). These findings suggest favorable predictive biomarkers for response to immunotherapy.

### 2.5. Treatment and Follow-Up

Due to impaired kidney function at the time, the patient did not receive neoadjuvant chemotherapy following a pathological diagnosis of lymphoepithelioma-like carcinoma (LELC) on the TURBT specimen. The tumor exhibited PD-L1 expression with a CPS of 90% by IHC as mentioned above, indicating favorable factors for immunotherapy response. Following the radical cystoprostatectomy, the patient received adjuvant immunotherapy with Pembrolizumab, an immune checkpoint inhibitor, for a year, completing 9 cycles. He underwent 3 years of clinical follow-up, during which multiple imaging studies (chest-abdominal-pelvis CT) showed no evidence of disease up to the time of this report.

## 3. Discussion

LELUC is a rare variant of UC, first reported in 1991 [3]. It accounts for 1% of bladder carcinomas and predominantly affects males in late adulthood [3, 4]. While nasopharyngeal and other tissue lymphoepitheliomas are associated with EBV, LELUC has consistently shown no association with EBV [5]. This histology is named after its resemblance to nasopharyngeal lymphoepithelioma [3]. In the current case, the tumor invades the muscularis propria and consists of sheets of undifferentiated cells with a characteristic syncytial appearance amidst a background of lymphoplasmacytic infiltrate. The tumor stains positive for PanCK, and rare tumor cells exhibit p63 and GATA3 staining. IHC for cytokeratin and urothelial markers highlights the epithelial cells and aids in distinguishing LELUC from high-grade lymphomas [5, 6]. In the appropriate diagnostic context, immunohistochemical markers such as GATA3, S-100P, CK7, CK20, high–molecular-weight cytokeratin (HMCK), and p63 can aid in confirming urothelial origin in tumors with variant histologic features [6]. These markers are typically not expressed in lymphomas, thereby assisting in distinguishing LELC of the urinary tract from hematolymphoid malignancies. The frequent detection of abnormalities by UroVysion FISH, the presence of UC in situ, and p53 overexpression by IHC in urinary tract LELC cases point to a pathogenesis closely aligned with that of high-grade invasive UC. Notably, unlike conventional UC, CK20 expression is often absent in LELC [5].

It has been suggested to divide the LELUC variant into subgroups: pure (where the variant constitutes 100% of the carcinoma), predominant (50%–100%), and focal (< 50%). Tumors with pure or predominant LELC features are associated with better overall survival due to known sensitivity to chemotherapy compared to those where LELC is mixed with conventional and other subtypes [2, 7, 8]. Our case had predominant LELC (95%) with a high TMB by molecular study and PD-L1 expression (TPS of 80% and CPS of 90%) by IHC. Our patient received adjuvant immunotherapy with an immune checkpoint inhibitor, completing 9 cycles of Pembrolizumab over the course of a year. Clinical follow-up demonstrated disease-free survival of 3 years to the time of this report. Our findings are supported by a recently reported study suggesting the potential use of immune checkpoint inhibitors as a therapeutic option for LELC tumors with PD-L1 expression [9].

Pure PUC is an exceedingly rare variant of UC that tends to present at an advanced stage with a poor prognosis. Similar to the LELUC variant, PUC was first reported in 1991 [3, 10]. This aggressive subtype is characterized by single infiltrating cells with a plasmacytoid appearance. The tumor cells mimic plasma cells or other inflammatory cells and resemble signet-ring carcinoma cells but lack extracellular mucin [10, 11]. The microscopic examination of our case showed that 5% of the UC comprised PUC. The PUC component consisted of discohesive tumor cells with signet ring cell morphology and aggressive features. The tumor penetrated through the muscularis propria and spread to the perivesical soft tissue surface and ureteral mucosa. During the examination of frozen sections, special attention should be given to the frequent positive surgical margins that lack grossly evident mass-like lesions, particularly in soft tissue surrounding ureteral margins [12, 13]. The majority of PUCs are positive for CD138, a plasma cell marker, and show loss of E-cadherin expression [14]. In the current case, the tumor cells showed loss of E-cadherin expression and were negative for CD138 by IHC. Differentiating PUC from nonurothelial tumors with plasmacytoid morphology, such as bladder metastasis from breast and stomach cancers or melanoma, can be challenging, and IHC can play an important role in the differential diagnosis [15–18]. For example, both PUC and metastatic lobular breast carcinoma can display GATA3 expression and loss of E-cadherin expression, which presents a differential diagnostic challenge. Markers such as ER, PR, mammaglobin, and TRPS1 are useful in these cases [16, 17, 19]. In differentiating PUC from gastric carcinomas, particularly gastric signet-ring cell carcinoma, certain histochemical and immunohistochemical tools are useful. Signet-ring cells in gastric carcinoma produce mucin, which can be demonstrated using mucicarmine staining. Additionally, these gastric tumor cells often exhibit positivity for CDX2, a marker indicative of gastrointestinal origin. When considering melanoma in the differential diagnosis of PUC, a panel of melanocytic markers, including S-100, Melan-A, HMB-45, and SOX10, is typically employed. The expression of these markers supports a diagnosis of melanoma and aids in distinguishing it from urothelial neoplasms with plasmacytoid morphology.

Although PUC does not yet have a standard definitive diagnosis protocol, markers including GATA3, CD138, Uroplakin II, Rb (loss), E-cadherin (loss), and additional molecular approaches such as UroVysion fluorescence in situ hybridization and FGFR3 mutational analysis can contribute to a potentially more accurate diagnosis [17, 18, 20].

In addition to the two UC variants, LELUC and PUC, the patient in the current case presented with a small percentage of early-stage prostatic adenocarcinoma. The incidental finding of prostate cancer (PCa) in radical cystoprostatectomy for UC is not uncommon. Many retrospective studies of patients with muscle-invasive bladder UC have found that the incidence of coexisting PCa is approximately 20% [21–23]. These PCas were either organ-confined or showed extraprostatic extension [21–23]. The majority of these PCas, approximately 90%, were limited to the organ (stage pT2), while approximately 10% were identified at a more advanced local stage (pT3 or higher) [21, 22].

Genomic profiling of the TURBT specimen of this case revealed several recurrent somatic mutations commonly associated with UC, including alterations in *TP53, TERT, RB1*, and *ARID1A*, as detailed in Table 2. These mutations are not specific to any particular histologic subtype of UC. The affected genes are involved in diverse cellular processes and key oncogenic pathways implicated in bladder cancer pathogenesis [24, 25]. The tumor suppressor genes *TP53* and *RB1* play central roles in regulating the cell cycle, while *ARID1A* is linked to chromatin remodeling and epigenetic control. Alterations in the *TERT* promoter represent the most frequently observed molecular event in UC. These mutations are present across all tumor grades and stages, as well as among various morphologic variants, yet are absent in nonneoplastic urothelium [24].

Additionally, structural changes involving the *GRIN2A* gene, such as amplifications and translocations, have been previously documented in bladder cancer [25]. In this case, a loss-of-function (stop-gain) mutation in *GRIN2A* was identified. This particular variant has not been reported in the existing literature based on a PubMed search, and its clinical or biological relevance remains uncertain.

Somatic mutations in *CDH1*, which often result in reduced or absent E-cadherin expression, are recognized as a molecular hallmark of the plasmacytoid variant of UC [24]. In our patient, only a minor component (approximately 5%) of plasmacytoid differentiation was observed in the cystoprostatectomy specimen, while it was absent in the TURBT sample submitted for molecular analysis. This may account for the lack of detectable *CDH1* mutations in this case.

In conclusion, a histologic variant of UC can be mixed with conventional and other subtypes, as well as with concomitant prostatic adenocarcinoma. The combination of LELUC and PUC is extremely rare, given that each subtype is uncommon on its own. A search of PubMed revealed no similar cases in the literature that discuss the clinicopathology and prognosis of simultaneous LELUC and PUC with collision of prostatic adenocarcinoma as described here. The predominant LELUC in the current case has a favorable prognosis, with a response to the immune checkpoint inhibitor, and no evidence of disease for 3 years up to the time of this report. PUC usually has a poor prognosis. The findings of a small percentage of PUC with clear resection margins may not affect the prognosis significantly in this case, but long-term clinical follow-up is necessary to support this assumption. Overall, our report demonstrates that the morphologic recognition of the various subtypes of UC, with the aid of IHC and molecular studies, is imperative for the proper and specific clinical management of patients.

## Figures and Tables

**Table 1:** Clones of antibodies utilized in immunohistochemistry (all monoclonal antibodies from the vendor of Roche).

**Antibody**	**PanCK**	**Gata3**	**P63**	**E-cadherin**	**CD138**
Clone	AE1/AE3/PCK26	L50-823	4A4	36	B-A38(MI15)

**Table 2:** Somatic genomic alterations of biological significance detected in the tumor tissue from the TURBT specimen, presented in order of decreasing variant allele fraction.

**Biologically relevant genomic variants (somatic)**	**Mutation effect**
TP53	p.P278S missense variant—LOF (loss of function)
TERT	c.-124C>T variant—Promoter mutation
RB1	p.R787^a^ stop gain—LOF
ARID1A	p.R750^a^ stop gain—LOF
TP53	p.Q331^a^ splice region variant—LOF
GRIN2A	p.E1175^a^ stop gain—LOF

^a^The presence of a stop codon.

## Data Availability

The data that support the findings of this study are available on request from the corresponding author. The data are not publicly available due to privacy or ethical restrictions.
